# Regional Disparities of Rehabilitation Resources for Persons with Disabilities in China: Data from 2014 to 2019

**DOI:** 10.3390/ijerph17197319

**Published:** 2020-10-07

**Authors:** Qi Jing, Qi Tang, Mei Sun, Xiaohong Li, Gang Chen, Jun Lu

**Affiliations:** 1 School of Public Health, Fudan University, Shanghai 200032, China; jingq@wfmc.edu.cn (Q.J.); tangqi@fudan.edu.cn (Q.T.); sunmei@fudan.edu.cn (M.S.); lixh@fudan.edu.cn (X.L.); gchen@shmu.edu.cn (G.C.); 2 School of Management, Weifang Medical University, Weifang 261053, China; 3 China Research Center on Disability Issues, Fudan University, Shanghai 200032, China; 4 Key Laboratory of Health Technology Assessment, National Health Commission, Fudan University, Shanghai 200032, China; 5 China Rehabilitation and Health Institute, Weifang Medical University, Weifang 261053, China

**Keywords:** disparity, rehabilitation resource, disability, HRDI, Theil Index, China

## Abstract

Although the United Nations’ Convention on the Rights of Persons with Disabilities enshrines the right to health for all persons with disabilities (PDs), PDs face health disparities in terms of access to rehabilitation resources, which is important for service supply. This study aimed to explore the trends and distribution of rehabilitation resources for PDs in China from 2014 to 2019, explore the main factors that influence equity, and provide suggestions for policymakers. Data were obtained from the annual China Statistical Bulletin on the Development of Disabled Persons and the database of the China Disabled Persons’ Federation. Six types of rehabilitation resources were chosen to measure the trends in allocation and equity. Data on disparities were analyzed based on western, central, and eastern regions. The Health Resource Density Index and Theil Index were calculated to determine the degree and density of unfairness. The findings show a steady increasing trend in the amount of rehabilitation resources in China from 2014 to 2019. The density and equity of allocation of rehabilitation resources have improved greatly in recent years. Regional disparities were principally caused by differences within the regions. Suggestions including expanding investment in rehabilitation resources and developing rehabilitation systems were put forward.

## 1. Introduction

It is estimated that approximately 15% of the global population has experienced some form of disability, and that between 110 and 190 million people have a severe physical impairment [1]. With the aging of the global population, as well as the prevalence of noncommunicable diseases and unhealthy lifestyles, the number of groups with disabilities will further increase [2,3]. Rehabilitation is a fundamental health service for people with a variety of health conditions, and it addresses the impact of a health condition on a person’s life by focusing primarily on improving their functioning and reducing the experience of disability [4]. Sufficient rehabilitation resources and their rational allocation are one of the foundations ensuring the achievement of the United Nation’s Sustainable Development Goal 3 (SDG): Ensure healthy lives and promote well-being for all at all ages, universal health coverage, and Rehabilitation 2030—Call for action [5]. This goal not only guarantees the fair treatment of the population, including persons with disabilities (PDs), but also follows the *Healthy China Strategy 2030*, which proposes to “enable all people to have needed, quality, and affordable health services such as prevention, treatment, rehabilitation, and health promotion.” This was particularly apparent during the 2020 COVID-19 pandemic, as the UN proposed *A Disability-Inclusive Response to COVID-19* to address the specific issues faced by PDs [6]. However, further prioritization of rehabilitation is urgently needed to meet the needs of PDs globally. There exists a global consensus to strengthen rehabilitation in health systems, which includes developing rehabilitation resources.

The United Nations’ Convention on the Rights of Persons with Disabilities (CRPD) enshrines the right to health for all PDs. All persons have the right to enjoy the highest attainable standard of health without discrimination on the basis of disability, and to have access to high-quality health services, including rehabilitation [7]. Often, however, PDs face health disparities in areas such as service availability, affordability, and quality of physical accessibility of resources, reflecting broader issues of disability inclusion. Furthermore, in our opinion, PDs from low- and middle-income countries should be given more attention due to their poor economic circumstances and vulnerability. As the world’s second largest economy, China is still a middle-income country with regional disparities relevant to specific economic and social development [8]. To improve the accessibility and availability of rehabilitation services for PDs, the government should take measures to allocate the health or rehabilitation resources that address the political commitment of social equity.

As the aging of China’s population continues, the prevalence of chronic diseases and the total number of PDs increase; thus, the demand for rehabilitation is growing rapidly. By the end of 2019, China’s elderly population (aged 60 and above) had reached 254 million, accounting for 18.1% of the total population of China [9] and 24.3% of the world’s elderly population, ranking first in the world (calculated by the authors). According to the China Disabled Persons’ Federation (CDPF) database, the number of registered persons with disabilities (RPDs) in China, which are categorized into four levels (Grades 1–4, with Grade 1 representing the most severe disability), reached more than 36.8 million in 2019. Under the Sixth National Population Census and the second national sample survey of PDs, it was estimated that the actual total number of PDs in China had reached 85 million, of whom nearly 50 million had rehabilitation needs [10].

Growing interest in inequality has brought many discussions on the subject into the public realm. However, few studies concentrate on rehabilitation resource disparities for PDs. Instead, the research has deeply explored the following three topics: (1) specific rehabilitation issues such as cardiac rehabilitation [11], exercise-based rehabilitation for non-communicable diseases [12], community-based rehabilitation resources [13], etc.; (2) health resource disparities including the perspectives of urban and rural communities [14], specific topics such as workforce [15] (physicians [16]), facilities [17] (institutions [18]), services, etc.; and (3) research methods on disparity or equity, for example the Theil Index [19], Gini-coefficient [20], Atkinson index [21], etc. Owing to this lack of academic concern for rehabilitation resource disparities for PDs, we propose the following research question: What are the trends, situations, disparities, and main influencing factors regarding rehabilitation resources for PDs in China? As the Theil Index is more sensitive in evaluating the equality of resource allocation, we aimed to explore the trends and distribution of rehabilitation resources for PDs in China from 2014 to 2019. Further, we investigated the main factors that influence equity or disparity and offer suggestions for policymakers. The data resources are the annual China Statistical Bulletin on the Development of Disabled Persons and the CDPF database. Six indicators were chosen to measure the trends in equity of rehabilitation resource allocation in China: Rehabilitation Institutions (RIs), Staff in RIs, Professionals in RIs, Total Completed Projects of Rehabilitation Service Facilities (RSFs), Total Construction Area of Projects Completed on RSFs, and the Total Investment of Projects Completed on RSFs. Regional disparities were analyzed by comparing the western, central, and eastern regions using the Theil Index and Health Resource Density Index (HRDI).

## 2. Materials and Methods

This study aimed to explore the trends and distribution of rehabilitation resources for PDs in China, explore the main factors that influence equity, and provide suggestions for policymakers. Then, the results may draw more attention to rehabilitation resource disparity. Regarding the limitation that the national statistics system does not include specific rehabilitation resources and study purposes, the rehabilitation resources data mainly come from the CDPF, which is a non-governmental and a national umbrella organization for persons with diverse disabilities (mainly visual, hearing, speech, physical, intellectual, and mental disabilities). The rehabilitation resources excluded medical resources such as hospitals, medical facilities, and workforce. In addition, it is estimated that the actual number of PDs is much higher than the RPDs who are registered with CDPF. To ensure a comparative analysis, this research used the official statistics of RPDs. Based on to data availability, the national analysis period was 2014–2019 and the regional analysis period was 2014–2018.

### 2.1. Data Resources and Regional Division

In this study, we collected the relevant data from the annual China Statistical Bulletin on the Development of Disabled Persons (2014–2019) and the CDPF database (2014–2018). All data are openly accessible on the CDPF’s public website. To analyze regional disparities, the 31 provinces, autonomies, and municipalities of mainland China were divided into three regions. According to geographical position and gross domestic product per capita, the three regions are described as western, central, and eastern, which represent undeveloped, developing, and developed regions in mainland China, respectively. Beijing, Tianjin, Hebei, Liaoning, Shanghai, Jiangsu, Zhejiang, Fujian, Shandong, Guangdong, and Hainan belong to the eastern region. Shanxi, Jilin, Heilongjiang, Anhui, Jiangxi, Henan, Hubei, and Hunan belong to the central region. Inner Mongolia, Guangxi, Chongqing, Sichuan, Guizhou, Yunnan, Tibet, Shaanxi, Gansu, Qinghai, Ningxia, and Xinjiang belong to the western region.

### 2.2. Measurements (Indicators)

Generally, health resource-related indicators include three basic aspects: workforce, financing, and property. After scanning the database, we chose six indicators: Staff and Professionals in RIs to represent the rehabilitation workforce; Total Completed Projects and Total Investment of Projects Completed on RSF to represent financing; and Rehabilitation Institution and Total Construction Area of Projects Completed on RSF to represent property. Then, we described and analyzed the six indicators. The indicator descriptions are as follows: (1) Staff in RIs refers to the persons who work at rehabilitation institutions, including professionals (rehabilitation physicians, nurses, therapists, technicians, etc.), managerial personnel (responsible for management and coordination), and other support personnel; (2) Total Completed Projects on RSFs refers to tailored construction and services projects, such as the establishment of specific rehabilitation training centers, and Total Investment of Projects Completed on RSFs refers to the annual investment on projects; and (3) Rehabilitation Institutions refers to institutions for different types of disabilities, such as visual, hearing and speech, physical, intellectual, mental, and autism in children. In this study, we included assistive technology institutions in RIs in statistics. Total Construction Area of Projects Completed on RSFs refers to the scale of the construction for projects completed on RSFs yearly.

### 2.3. Main Analysis Methods

#### 2.3.1. Health Resource Density Index

HRDI was used to assess equity both in demographic and geographic dimensions. The HRDI was combined with the data to demonstrate differences in rehabilitation resources for PDs in 31 provinces and municipalities. In addition, the HRDI could avoid bias and influences caused by a single aspect of population or geographic area [22]. The value of HRDI equals the geometric mean of rehabilitation resources per 10,000 RPDs and per 10,000 square kilometers. The formula of HRDI is as follows:

(1) $$HRDI=\sqrt{\left(r_{i}/p_{i})\times (r_{i}/a_{i}\right)},$$

In the above formula, *r_i_* represents the rehabilitation resource, *p_i_* represents RPDs, and *a_i_* represents the square of the unit *I*.

#### 2.3.2. Theil Index

The Theil Index, named for the researcher who first proposed it in 1967 [23], is an inequality measure related to Shannon entropy and a relative indicator that ranges from 0 to 1. A low Theil value means low inequality, while high values represent a high deviation from an equal distribution. A Theil Index of 0 indicates perfect equality and that every region has the correct proportion of resources for the population. Conversely, a standardized Theil Index value of 1 represents a state of perfect inequality, where one region has all the variables of interest. The Theil Index has been tested in a series of areas, such as the economic [24], energy [25], and health sectors [26], to evaluate equity and disparity. The formula of the Theil Index is as follows:

(2) $$T=\sum_{i=1}^{n}p_{i}\ln\frac{p_{i}}{y_{i}}.$$

where *p_i_* represents the proportion of the unit RPDs accounting for the aggregation in one region and *y_i_* represents the unit’s rehabilitation resource accounting for the aggregation in one region.

The Theil Index can be decomposed into two components: to describe inequality “within” and “in between” subgroups. The formulas are as follows:

(3) $$T_{w}=\sum_{g=1}^{k}p_{g}t_{g},$$

(4) $$T_{b}=\sum_{g=1}^{k}p_{g}\ln\frac{p_{g}}{y_{g}},$$

(5) $$T=T_{w}+T_{b}.$$

T_w_ represents the degree of rehabilitation resource allocation fairness in a targeted region; T_b_ represents the degree of rehabilitation resource allocation equity between the different regions; and *p_g_* and *y_g_* have the same meaning as *p_i_* and *y_i_* above.

## 3. Results

### 3.1. Trends of Rehabilitation Resource Allocation from 2014 to 2019

Changes in the amount of rehabilitation resources for PDs are shown in Table 1. Generally, the rehabilitation resources for PDs in China have been increasing in recent years. The RPDs figure grew from 29.47 million to 36.82 million, and the rehabilitation resources of the six indicators per 10,000 RPDs generally increased from 2014 to 2019. The total investment and construction area of projects completed on RSFs for PDs, which increased in 2019, were triple those of 2014. Per 10,000 RPDs, the number of RIs, staff, professionals, and completed projects rose 41.38%, 13.00%, 18.75%, and 64.11%, respectively. In terms of density, the workforce for rehabilitation per 10,000 RPDs decreased in 2019 when compared to 2014 (staff, −9.55%; professionals, −4.95%).

### 3.2. Regional Distribution of Rehabilitation Resources for PDs in 2018

To further understand the rehabilitation resource distribution in China, we calculated the rehabilitation resources per 10,000 RPDs and per 10,000 km^2^ in 2018 (the provincial data have been updated only to 2018). The results are shown in Table 2 and Table 3, respectively. Generally, when compared to other provinces, eastern areas such as Shanghai, Guangdong, and Shandong, central areas including Shanxi, and western areas such as Chongqing had higher distribution values, both per 10,000 RPDs and geographically on all types of rehabilitation resources.

From the perspective of per 10,000 RPDs, we could acquire a basic understanding of provincial resource distribution and density. In terms of the rehabilitation institutions for PDs, the majority of the values were concentrated below 5, except for Shanghai, which reached almost 19. Guangdong had the highest value of staff and professionals in distribution of RIs with more than 100 persons per 10,000 RPDs. Additionally, we found extreme disparities between the highest and lowest values: the number of RIs per 10,000 RPDs in Shanghai (18.96) was 30 times higher than that in Tibet (0.58), while the eastern regions were 1.8 times higher than the western regions.

Regarding the aspect of regional distribution per 10,000 km^2^, the resources were concentrated in five eastern areas (Shanghai, Beijing, Tianjin, Guangdong, Jiangsu, and Shandong), all of which ranked among the top 10 in almost all six indicators. The geographic distribution gap was larger than that of the demographic. For the other indicators, and apart from the projects of RSFs for PDs, Shanghai was the highest ranked and Tibet was the lowest.

### 3.3. Regional HRDI of Rehabilitation Resources for PDs in 2018

To avoid the bias and influences caused by a single aspect of population or geographical area, HRDI was applied to assess equity both in demographic and geographic dimensions. We calculated formula (1), which indicated that the 31 provinces and municipalities had large regional disparities. The highest HRDI in all indicators was at least 30 times higher than the lowest. The largest disparity regarding HRDI was evident within the RI indicator. Geographically, the eastern regions’ HRDI was 4.2 and 1.8 times higher than those of the western and central areas, respectively. Shanghai ranked first in five indicators, while Fujian ranked first in one indicator. Tianjin, Guangdong, Beijing, Jiangsu, Chongqing, Liaoning, Shandong, Shanxi, etc. ranked within the top 15 HRDI.

### 3.4. Theil Index of Rehabilitation Resources Allocation Based on RPDs

**Figure 1:**
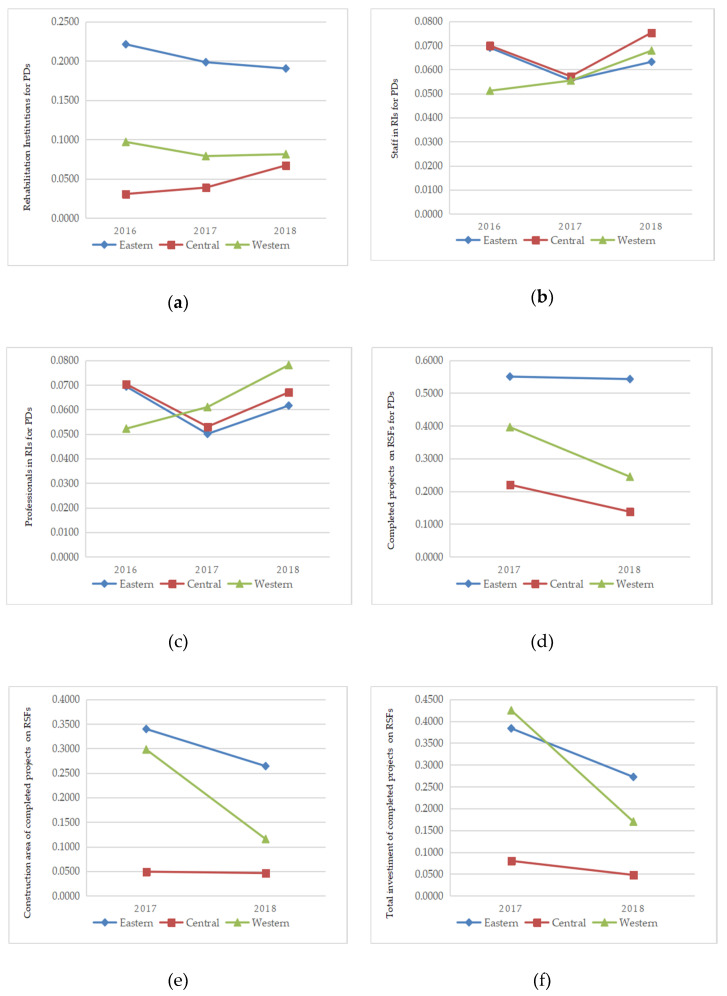
The regional Theil Index trends of six types of rehabilitation resources (which showed from (**a**) to (**f**) respectively) in China from 2016 to 2018. (**a**) Theil index values of RIs for PDs from 2016 to 2018 in the three regions; (**b**) Theil index values of staffs in RIs for PDs from 2016 to 2018 in the three regions; (**c**) Theil index values of professionals in RIs for PDs from 2016 to 2018 in the three regions; (**d**) Theil index values of completed Projects on RSFs for PDs from 2017 to 2018 in the three regions; (**e**) Theil index values of construction area of completed projects on RSFs from 2017 to 2018 in the three regions; (**f**) Theil index values of total investment of completed projects on RSFs from 2017 to 2018 in the three regions.

The results shown in Table 2, Table 3 and Table 4 present the context of provincial rehabilitation resources for allocation to PDs, from which we noticed a large difference between the regions. Generally, the share of rehabilitation resources in the eastern regions is more than that in the central and western regions. During further investigation on the extent of the regional disparities, the Theil Index was calculated in the three regions from 2016 to 2018 (Figure 1). Excluding the data regarding staff and professionals, the regional data of the remaining four indicators were changed or missing. The Theil Index indicated that the data on RIs, staff, and professionals were from 2016 to 2018, and that the other three indicators were from 2017 to 2018. The Theil Index of projects of RSFs was significantly higher from 2016 to 2018 than those of other kinds of resources. In 2018, it decreased from approximately 0.55 to 0.43, which was still higher than those of RIs (0.15), staff (0.09), professionals (0.09), construction area (0.20), and investment of the projects (0.25). Excluding the staff and professionals, the Theil Index of all the rehabilitation resources showed a general downward trend in the most recent three years. The whole Theil Index was divided into three regions. The following figure could reflect the changes of the Theil Index during 2016–2018.

The Theil Index of the eastern regions showed a higher value than the central or western regions. In terms of the rehabilitation workforce, the Theil Index of staff and professionals in RIs presented a first decreasing and then increasing trend (both 0.09 in 2018), but their values were obviously lower than those of the other four indicators, which were more than 0.15. Apart from the five other indicators, the western region’s Theil Index for professionals in RIs for PDs was higher than that of the central and eastern regions: the western region was the least equitable with regard to workforce distribution. Compared to personnel, fairness in the allocation of other rehabilitation resources clearly improved as the values decreased.

As shown in Table 5, the between-group contribution rates were less than 35% in every indicator and year, they and were lower than the proportion of within-group contribution rates. Except for the investment of completed projects in RSFs for PDs, the between-group contributions of other rehabilitation resources showed a decreasing trend. That is, the main contribution for the total Theil Index, which represented equity, was the within-group Theil Index.

## 4. Discussion

Rehabilitation is a fundamental health service for people with a variety of health conditions, and primarily focuses on improving their ability to function well in society and on reducing the impact of disability. This was a nationwide study that comprehensively evaluated the trend of rehabilitation resources allocation tailored for PDs in China. We found a steady rising trend in the total amount of rehabilitation resources in China, annually per 10,000 RPDs and per 10,000 km^2^. Although the United Nations’ CRPD enshrines the right to health for all PDs, PDs face health disparities in terms of access to rehabilitation services. However, our findings indicate that the opportunities for PDs to obtain rehabilitation services are expanding. With the development of the social economy, the aging of the population, and an increase in both the number of groups with disabilities and the various public health initiatives, there has also been an increase in the awareness of not only the general public but also the government of opportunities within the rehabilitation sector. This trend is clearly related to the rapid development of both China’s social economy and its medical reform policies.

The growth rate of the total rehabilitation workforce was lower than that of other selected rehabilitation resources. Furthermore, the personnel density of rehabilitation institutions for PDs has decreased by almost 10% compared to five years ago. Together, these data indicate that the focus on rehabilitation for PDs may be more concentrated on property, infrastructure, and projects, particularly as investment and rehabilitation resources have increased significantly. In addition, because of the new round of health system reforms enacted in 2009, the growth in health spending has also increased rapidly to support the development of the rehabilitation sector.

This study explored trends, density, distribution, and equity analysis using data from 2014 to 2019 with data from the CDPF. Based on the results of this study, we found that the overall fairness of rehabilitation resource allocation has improved in China. However, further results for the distribution of rehabilitation resources per 10,000 RPDs and per 10,000 km^2^ show that there was a large gap between different provinces and regions. Furthermore, there was a larger disparity in the geographic distribution of rehabilitation resources than in the distribution of RPDs. With respect to existing research on health resources [27], this study is unique, as few studies are concerned with this specific area of research.

The value for staff or professionals of rehabilitation institutions showed a downward trend regarding density (both per 10,000 RPDs and per 10,000 km^2^), and the increasing rates were lower than RPDs. There are three reasons for this finding. First, compared to international training of rehabilitation professionals, China only started systematic rehabilitation education after 2001, and the educational level was mainly focused on vocational or college education. The university major of rehabilitation therapy was not divided into specific professional directions, such as physical therapy, occupational therapy, and speech therapy. Thus, the rehabilitation workforce entering the job market is limited, including rehabilitation institutions for PDs. Second, there is no professional certification system; professionals, such as rehabilitation therapists, cannot practice as medical practitioners. Third, the general public has a low perception of the occupation and regards it as similar to “massage”, leading to low career attraction and high turnover. However, the government has recognized these challenges by expanding higher education on rehabilitation [28]. The University of Rehabilitation is being established in Qingdao, Shandong province, eastern China. Indeed, the higher is the standardized education, the more attractive is the occupation.

The difference in the geographic and demographic density of rehabilitation resources will inevitably lead to an imbalance in rehabilitation resource access for PDs; therefore, we also calculated the HRDI to analyze the comprehensive density in each unit in 2018. The results indicate that there was a comprehensive gap between different provinces and municipalities. Shanghai ranked first in HRDI for all indicators, except for projects of RSFs for PDs. Although rehabilitation resources are related to economic development, it is possible that the concentration of the government or the regional Disabled Person’s Federation is more important: this would explain why some eastern provinces were low-ranking. Moreover, there were many more regional gaps and disparities among the three regions: the eastern region’s HRDIs of all indicators were on average 2.7 and 4.7 times higher than those of the central and western regions, respectively. At the same time, the central region was 1.9 times higher than the western region.

However, are the rehabilitation resources fair? The results of the Theil Index show that the national Theil Index value has been decreasing annually, which implied that there was better equity within China; however, the highest rate value was found in the projects of RSFs for PDs in 2017 (0.55). The results are not surprising given that the projects, construction area, and investment goals are relatively easy to achieve compared to workforces whose employees require a long training period. However, as the rehabilitation sector for PDs is to some extent a part of public welfare, rehabilitation resources should be allocated scientifically and without bias. Another finding indicates that the causes of inequality in the eastern, central, and western regions principally came from the within-group rather than between-group rate. Compared to health resources, the Theil Index values of rehabilitation resources were higher; however, health resources, such as doctors, experienced the same declining trend during the same time period [16]. The level of economic development and amount of rehabilitation resources in the eastern provinces, such as Shanghai, Beijing, Tianjin, Guangdong, Jiangsu, and Shandong, were much higher than in other provinces. There were more projects per 10,000 RPDs in Fujian (eastern region) and project investment per 10,000 RPDs in Ningxia (western region) than indicated in other areas. Another interesting phenomenon is that the Theil Index showed that the contribution rate among these groups was around 70–75%, thus indicating that unfairness is mainly due to the within-group rate.

As a result, policy makers should focus on the allocation of rehabilitation resources and guide policy in weak provinces in the central and western regions to achieve overall coordinated development for improving access to rehabilitation services for PDs. Our suggestions are as follows: First, the rehabilitation sector should receive more investment. Second, all levels of rehabilitation education should be expanded. Third, the rehabilitation-related statistics system should be further improved, including by inserting it into the national statistics system. Fourth, the government should motivate the market to boost careers in rehabilitation for PDs. Fifth, accreditation of education and regulation of the rehabilitation workforce should be standardized. Sixth, national and sub-national plans regarding the allocation of rehabilitation resources should be created or inserted into a multi-level plan, such as the 14th Five-Year Plan. Finally, the rehabilitation system should be developed and improved within the existing health system. Only in this way can we achieve the goals of Healthy China 2030, Universal Health Coverage, and of SDG3.

Although this study specifically described the data of rehabilitation resources from 2014 to 2019, there are some limitations of this data source. Due in part to the data content and indicators having changed in recent years, some data in this study were predicted and estimated, and, furthermore, provincial analysis was only available for a specific period of time. Thus, there is some undue influence on the results. In addition, to discuss the rehabilitation resources for PDs, we used RPDs as the population, weighted on density and the Theil Index analysis. The actual number of potential PDs may be far higher than the RPDs. Moreover, unlike hardware, such as investment and projects, which could be improved in a short period of time, human resources (i.e., the workforce) are key for rehabilitation services and require a longer period of time to be cultivated and trained. Therefore, our next study will continue to follow the updated data from the CDPF. The important results have inspired further analysis on the allocation of human resources for the rehabilitation of PDs, and the rehabilitation resources require evaluation: these areas could represent a future avenue for research.

## 5. Conclusions

The density and equity of rehabilitation resource allocation has improved markedly in recent years. Compared to the four other indicators in this study, staffs and professionals in RIs for PDs demonstrated lower density and equity in all regions. The distribution of accumulated projects completed of RSFs was found to be the most inequitable when compared to the other five types of rehabilitation resources. Regional disparities were typically caused by differences within the regions. As the population ages and the number of disabilities expands, the need for rehabilitation will only continue to increase. The inequality phenomenon might be caused by various factors; thus, we could not make perfectly accurate measurements and assessments of fairness via one kind of tool or unilateral indices. However, we hope that this study can draw attention to the rehabilitation aspect of PDs to improve their access to basic and necessary services.

## Figures and Tables

**Table 1:** Rehabilitation resources for PDs in China from 2014 to 2019 ^a^.

Year	Registered Persons with Disabilities	RIs for PDs		Staff in RIs		Professionals in RIs		Total Completed Projects of RSFs for PDs		Total Construction Area of Accumulated Projects Completed on RSFs for PDs		Total Investment of Projects Completed on RSFs for PDs	
	10,000 Persons	Number	per 10,000 RPDs	Number	per 10,000 RPDs	Number	per 10,000 RPDs	Number	per 100,000 RPDs	10,000 m^2^	m^2^ per RPDs	100,000,000 Yuan	Yuan per RPDs
2014 ^b^	2947.0	6914	2.35	233,631	79.28	160,000	54.29	613	2.08	130.01	0.04	40.38	137.02
2015	3145.7	7111	2.26	232,370	73.87	159,563	50.72	682	2.17	165.65	0.05	51.27	162.98
2016	3219.4	7858	2.44	222,594	69.14	149,446	46.42	762	2.37	213.40	0.07	65.70	204.08
2017	3404.0	8334	2.45	245,822	72.22	164,264	48.26	833	2.45	261.40	0.08	80.80	237.37
2018	3566.2	9036	2.53	250,469	70.23	176,471	49.48	914	2.56	344.90	0.10	111.20	311.82
2019 ^b^	3681.7	9775	2.66	264,000	71.71	190,000	51.61	1006	2.73	414.20	0.11	132.20	359.07

^a^ Abbreviations: RIs, Rehabilitation Institution; PDs, Persons with Disabilities; RPD, Registered Persons with Disabilities; RSFs, Rehabilitation Service Facilities (this information is identical for the following tables). ^b^ The data came from two resources. If the provincial database lacked the data, we chose the China Statistical Bulletin on the Development of Disabled Persons as the data source; however, the numbers may not be as accurate as the official website.

**Table 2:** Regional distribution per 10,000 RPDs of rehabilitation resources for PDs in China in 2018.

Region	Area	RIs for PDs	Staff in RIs	Professionals in RIs	Projects of RSFs for PDs	Construction Area of Projects of RSFs for PDs	Investment of Accumulated Projects of RSFs for PDs
National		2.53	70.23	49.48	0.26	967.24	311.69
Eastern	Beijing	2.41	71.76	49.28	0.57	251.38	157.31
Eastern	Tianjin	2.64	66.10	43.23	2.97	555.42	322.99
Eastern	Hebei	2.28	66.46	47.02	0.48	306.89	90.66
Eastern	Liaoning	3.55	91.49	64.53	1.89	932.42	224.51
Eastern	Shanghai	18.96	158.53	83.41	0.89	1778.26	798.96
Eastern	Jiangsu	2.68	84.18	59.70	3.94	1523.05	608.03
Eastern	Zhejiang	1.62	50.70	35.10	3.15	2159.14	859.03
Eastern	Fujian	2.96	67.51	45.80	23.68	680.43	181.08
Eastern	Shandong	2.49	98.48	76.39	4.68	2882.29	832.23
Eastern	Guangdong	4.75	161.04	114.65	2.98	1271.95	422.12
Eastern	Hainan	1.91	59.69	45.40	2.24	622.05	208.19
Central	Shanxi	3.39	101.49	75.37	4.36	1198.01	328.86
Central	Jilin	3.46	131.96	85.54	1.18	464.38	185.25
Central	Heilongjiang	2.89	62.84	45.30	0.89	646.21	189.15
Central	Anhui	1.33	32.60	25.00	1.18	690.37	247.82
Central	Jiangxi	1.88	53.51	33.22	1.16	1065.00	187.25
Central	Henan	1.41	50.14	39.41	0.88	595.06	137.87
Central	Hubei	1.39	47.28	31.46	0.91	445.25	137.85
Central	Hunan	2.32	70.20	50.03	2.08	563.40	124.32
Western	Inner Mongolia	2.57	61.81	39.79	2.32	1137.30	399.03
Western	Guangxi	2.68	64.32	45.92	0.74	479.01	126.29
Western	Chongqing	3.02	84.58	56.83	1.48	926.10	346.84
Western	Sichuan	1.01	38.57	26.02	1.67	750.78	320.68
Western	Guizhou	1.60	57.70	38.56	0.74	520.67	132.21
Western	Yunnan	1.84	49.73	34.64	0.50	306.64	85.89
Western	Tibet	0.58	6.12	3.79	12.62	2163.99	804.14
Western	Shaanxi	2.64	92.83	69.87	2.95	643.15	192.92
Western	Gansu	1.65	39.47	27.58	2.04	1051.00	254.01
Western	Qinghai	1.72	22.44	13.41	3.32	1875.73	752.88
Western	Ningxia	1.27	24.05	16.54	2.55	2176.88	887.83
Western	Xinjiang	3.15	56.71	40.00	5.68	1537.16	432.98

**Table 3:** Regional distribution per 10,000 km^2^ of rehabilitation resources for PDs in China in 2018.

Region	Area	RIs for PDs	Staff in RIs	Professionals inRIs	Project of RSF for PDs	Construction Area of Accumulated Projects of RSFs for PDs	Investment of Accumulated Projects of RSFs for PDs
National		9.41	260.91	183.82	0.95	3593.08	1157.86
Eastern	Beijing	77.44	2301.22	1580.49	1.83	8061.59	5044.94
Eastern	Tianjin	74.17	1856.67	1214.17	8.33	15600.00	9071.67
Eastern	Hebei	22.83	665.78	471.03	0.48	3074.21	908.16
Eastern	Liaoning	25.41	654.19	461.42	1.35	6666.96	1605.29
Eastern	Shanghai	1700.00	14215.87	7479.37	7.94	159463.49	71645.87
Eastern	Jiangsu	40.58	1275.84	904.76	5.97	23083.30	9215.32
Eastern	Zhejiang	20.24	632.02	437.52	3.93	26915.03	10708.42
Eastern	Fujian	22.57	514.33	348.93	18.04	5183.77	1379.54
Eastern	Shandong	35.72	1411.72	1095.06	6.71	41316.72	11929.78
Eastern	Guangdong	40.79	1384.03	985.31	2.56	10931.61	3627.86
Eastern	Hainan	9.60	300.85	228.81	1.13	3135.31	1049.35
Central	Shanxi	20.87	624.38	463.69	2.68	7370.64	2023.26
Central	Jilin	15.69	598.99	388.26	0.53	2107.90	840.88
Central	Heilongjiang	6.83	148.52	107.06	0.21	1527.40	447.08
Central	Anhui	16.92	413.99	317.56	1.50	8767.74	3147.35
Central	Jiangxi	12.58	358.42	222.53	0.78	7134.03	1254.34
Central	Henan	23.05	822.10	646.23	1.44	9757.31	2260.60
Central	Hubei	11.51	390.32	259.71	0.75	3675.47	1137.95
Central	Hunan	18.93	572.29	407.84	1.70	4593.06	1013.48
Western	Inner Mongolia	1.78	42.83	27.57	0.16	788.12	276.52
Western	Guangxi	15.28	366.16	261.41	0.42	2726.94	718.94
Western	Chongqing	32.16	899.88	604.61	1.58	9853.28	3690.23
Western	Sichuan	5.72	218.27	147.26	0.95	4249.03	1814.90
Western	Guizhou	11.01	398.07	266.00	0.51	3592.28	912.15
Western	Yunnan	6.52	175.82	122.46	0.18	1084.14	303.66
Western	Tibet	0.05	0.51	0.32	0.11	181.46	67.43
Western	Shaanxi	17.41	612.69	461.19	1.95	4244.99	1273.33
Western	Gansu	3.03	72.65	50.76	0.38	1934.56	467.54
Western	Qinghai	0.43	5.61	3.35	0.08	468.75	188.15
Western	Ningxia	4.52	85.39	58.73	0.90	7728.01	3151.84
Western	Xinjiang	1.10	19.80	13.96	0.20	536.65	151.16

**Table 4:** Regional HRDI of rehabilitation resources for PDs in China in 2018.

Region	Area	RIs for PDs	Staff in RIs	Professionals in RIs	Projects of RSF for PDs	Construction Area of Accumulated Projects of RSFs for PDs	Investment of Accumulated Projects of RSFs for PDs
National		4.88	135.37	95.38	0.49	1864.23	600.74
Eastern	Beijing	13.67	406.36	279.09	1.02	1423.55	890.86
Eastern	Tianjin	13.99	350.33	229.10	4.97	2943.56	1711.73
Eastern	Hebei	7.21	210.36	148.82	0.48	971.30	286.94
Eastern	Liaoning	9.50	244.65	172.56	1.60	2493.27	600.34
Eastern	Shanghai	179.52	1501.21	789.83	2.65	16839.47	7565.86
Eastern	Jiangsu	10.42	327.72	232.40	4.85	5929.34	2367.11
Eastern	Zhejiang	5.73	179.01	123.92	3.52	7623.20	3032.97
Eastern	Fujian	8.18	186.34	126.42	20.67	1878.08	499.81
Eastern	Shandong	9.43	372.87	289.23	5.61	10912.69	3150.93
Eastern	Guangdong	13.91	472.10	336.10	2.76	3728.86	1237.49
Eastern	Hainan	4.28	134.00	101.92	1.59	1396.54	467.41
Central	Shanxi	8.41	251.72	186.94	3.42	2971.55	815.70
Central	Jilin	7.36	281.14	182.24	0.79	989.38	394.68
Central	Heilongjiang	4.44	96.60	69.64	0.43	993.49	290.80
Central	Anhui	4.75	116.17	89.11	1.33	2460.29	883.17
Central	Jiangxi	4.86	138.48	85.98	0.95	2756.40	484.64
Central	Henan	5.69	203.02	159.59	1.12	2409.61	558.27
Central	Hubei	4.01	135.85	90.39	0.83	1279.25	396.06
Central	Hunan	6.63	200.43	142.84	1.88	1608.64	354.95
Western	Inner Mongolia	2.14	51.45	33.12	0.61	946.75	332.17
Western	Guangxi	6.40	153.47	109.56	0.56	1142.91	301.32
Western	Chongqing	9.86	275.88	185.36	1.53	3020.78	1131.34
Western	Sichuan	2.40	91.75	61.90	1.26	1786.08	762.89
Western	Guizhou	4.19	151.55	101.27	0.61	1367.63	347.26
Western	Yunnan	3.47	93.51	65.13	0.30	576.58	161.50
Western	Tibet	0.17	1.77	1.10	1.16	626.63	232.86
Western	Shaanxi	6.78	238.49	179.51	2.39	1652.32	495.63
Western	Gansu	2.23	53.55	37.42	0.88	1425.91	344.61
Western	Qinghai	0.86	11.22	6.70	0.53	937.68	376.37
Western	Ningxia	2.40	45.32	31.17	1.52	4101.58	1672.81
Western	Xinjiang	1.86	33.51	23.63	1.06	908.25	255.83

**Table 5:** Theil Index and contribution rates of rehabilitation resources for PDs in China.

Rehabilitation Resource	Year	Theil Index	Within-Group	Between-Group
RIs for PDs	2016	0.1795	65.90%	34.10%
	2017	0.1529	69.66%	30.34%
	2018	0.1532	74.29%	25.71%
Staff in RIs	2016	0.0921	68.75%	31.25%
	2017	0.0800	70.00%	30.00%
	2018	0.0921	74.64%	25.36%
Professionals in RIs	2016	0.1016	68.75%	31.25%
	2017	0.0825	70.00%	30.00%
	2018	0.0923	74.64%	25.36%
Projects of RSFs for PDs	2017	0.5454	71.73%	28.27%
	2018	0.4275	72.75%	27.25%
Construction area of completed projects of RSFs for PDs	2017	0.3388	68.09%	31.91%
	2018	0.1980	72.39%	27.61%
Investment of completed projects of RSFs for PDs	2017	0.4213	70.68%	29.32%
	2018	0.2471	66.51%	33.49%

## Abbreviations

SDGs	Sustainable Development Goals
PD	Persons with Disabilities
CRPD	Convention on the Rights of Persons with Disabilities
CDPF	China Disabled Persons’ Federation
RPD	Registered Persons with Disabilities
RI	Rehabilitation Institution
RSF	Rehabilitation Service Facility
